# Supplementary material for: The association of maternal factors with the neonatal microbiota and health
Source: Nat Commun. 2024 Jun 19;15:5260. doi: 10.1038/s41467-024-49160-w (PMC11187136; doi:10.1038/s41467-024-49160-w)
Supplement: Supplementary file 1 — Supplementary Information [file 41467_2024_49160_MOESM1_ESM.pdf]

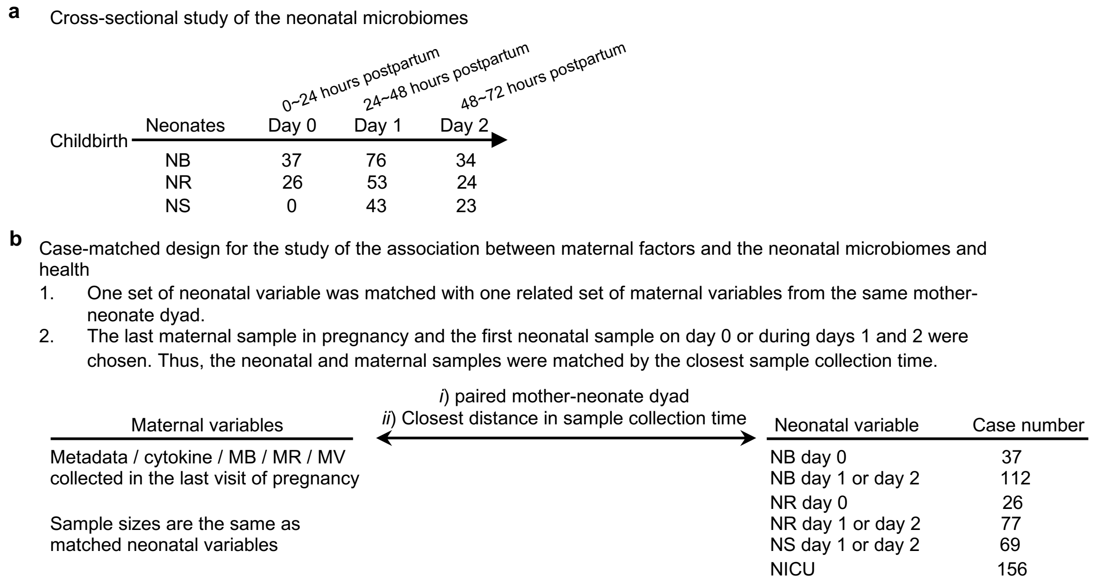

Fig. S1 Experimental design of the study. (a) Sample numbers in the cross-sectional study to determine the profiles of the neonatal microbiotas within three days postpartum. NB, NR, and NS are the neonatal buccal, rectal, and stool microbiotas, respectively. **(b)** A case-matched design to study the association between maternal factors and the neonatal microbiotas. Samples at the same time points are biological replicates.

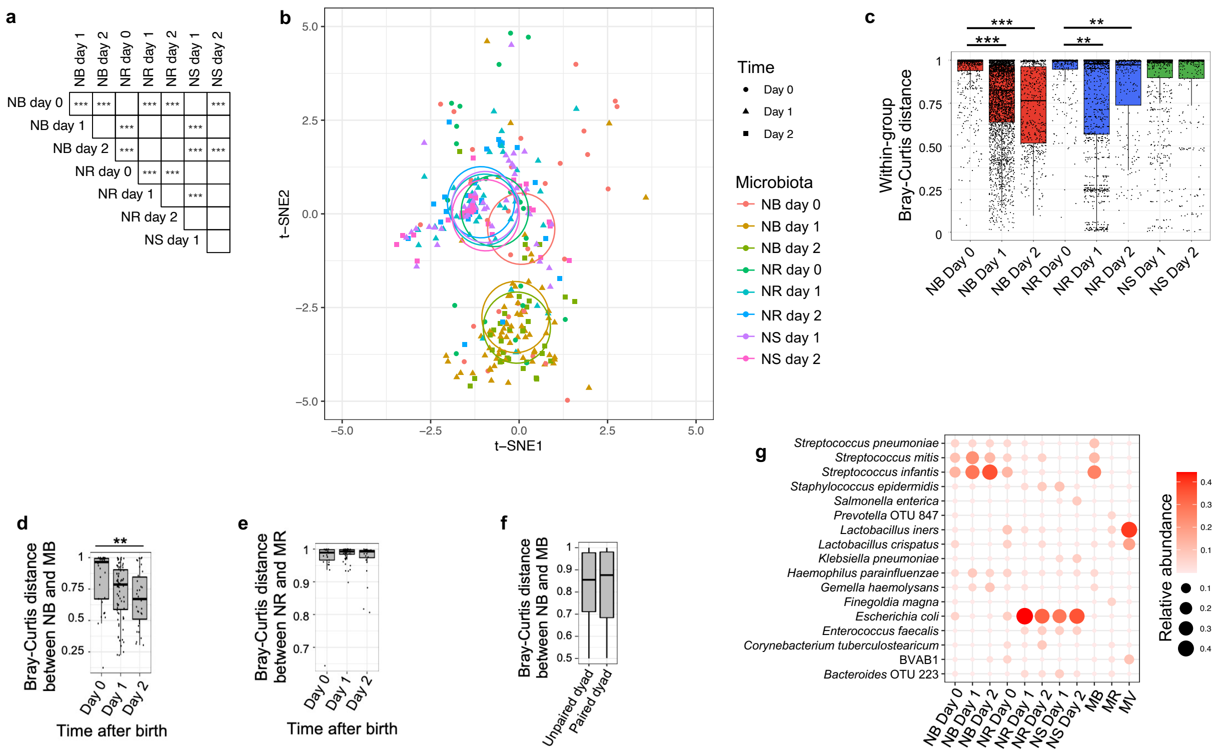

Fig. S2 Beta diversity and composition of the neonatal microbiotas within three days postpartum. (a) the PERMDISP test to determine the differences in dispersion between two microbiotas. **(b)** A t-SNE plot to show the centroids of the neonatal microbiotas. **(c)** Comparison of the within-group Bray-Curtis distance of the neonatal

microbiotas using the multiple response permutation procedure test. ** P-value ≤ 0.01, and *** P-value ≤ 0.001. The change in the Bray-Curtis distance between the NB and MB **(d)** and NR and MR **(e)** over time quantified by the two-sided Kruskal–Wallis test. ** P-value ≤ 0.01. **(f)** Comparison of the Bray-Curtis distance between paired and unpaired NB-MB microbiome dyads using the two-sided Mann–Whitney U test. **(g)** The relative abundance on average of the top three abundant taxa in any studied microbiome. Lines in the boxplots represent maximum, 75% quantile, median, 25 quantile, and minimum values from top to bottom.

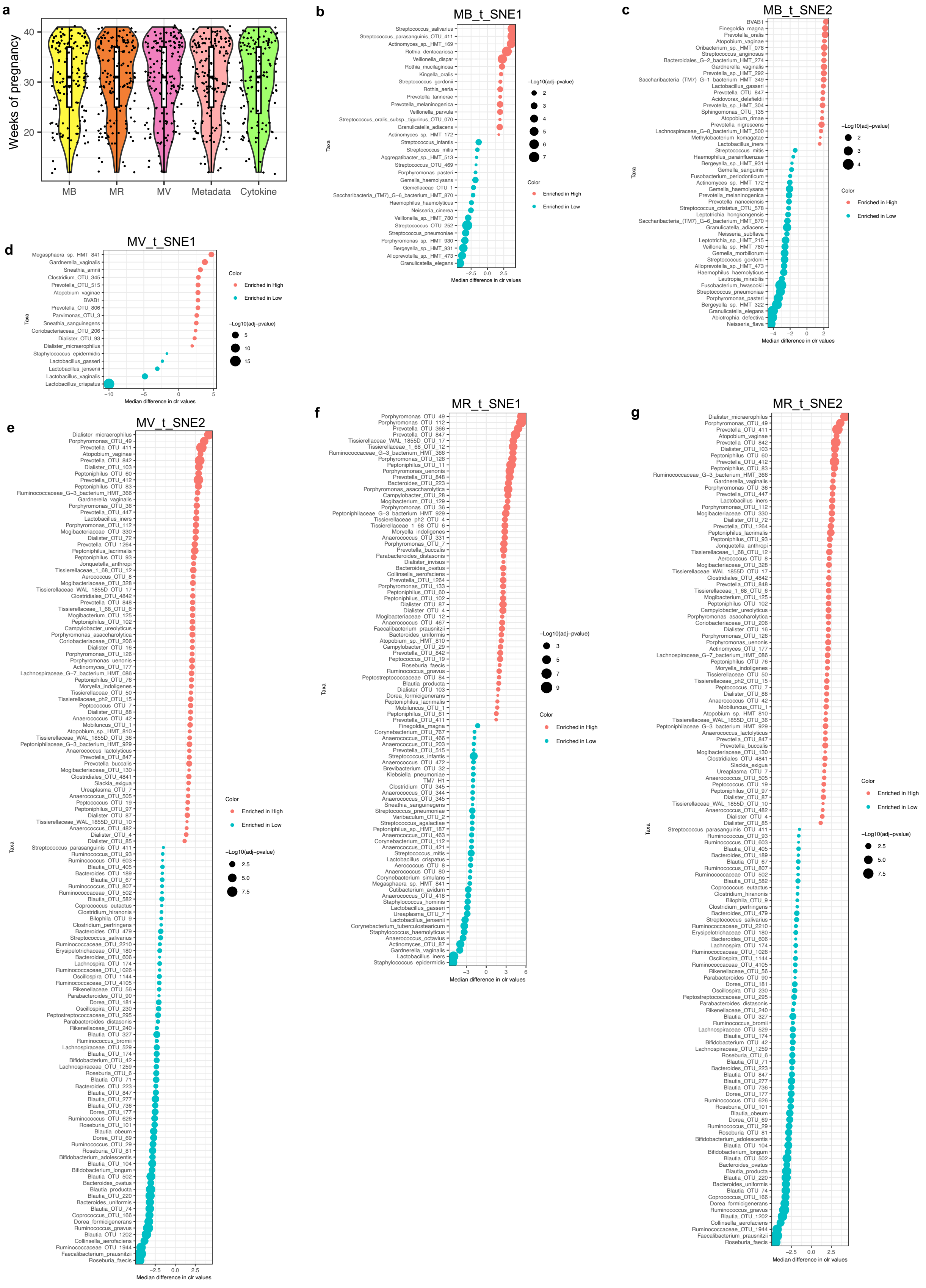
a
Weeks of pregnancy
MB
MR
MV
Metadata
Cytokine
b
MB_t_SNE1
c
MB_t_SNE2
d
MV_t_SNE1
e
MV_t_SNE2
f
MR_t_SNE1
g
MR_t_SNE2
Taxa
Median difference in clr values
−Log10(adj−pvalue)
Color
Enriched in High
Enriched in Low

Fig. S3 Maternal data collection time and biological meaning of the t-SNE values of the maternal microbiotas. (a) Data collection time for the MB, MR, and MV microbiotas, maternal metadata, and cytokine data. The difference was determined by the two-sided Mann-Whitney U test and no significant difference was detected. Lines in the boxplots represent maximum, 75% quantile, median, 25 quantile, and minimum values from top to bottom. **(b-g)** The biological meaning of the t-SNE1 and t-SNE2 values in the maternal microbiotas was illustrated by testing the abundance differences between the microbiotas with high and low levels of the t-SNE values.

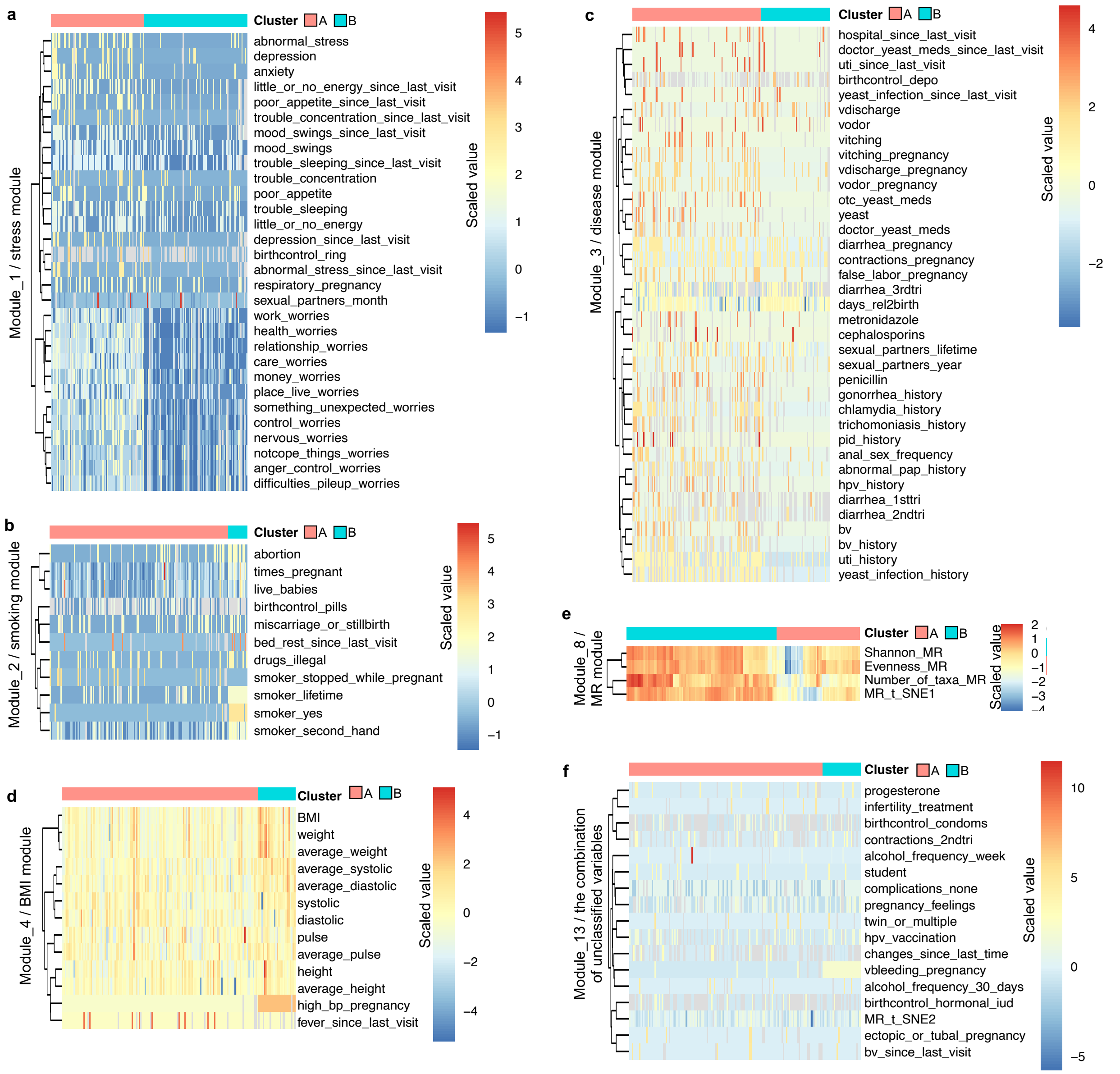

Fig. S4 The classification of the 164 maternal-neonatal dyads according to maternal factors in each module. The 164 maternal-neonatal dyads were classified to groups A and B according to the clustering of the Gower's distance of maternal variables in each maternal factor module. The results of modules 1, 2, 3, 4, 8, 9, 12, and 13 are shown on a-h, respectively.

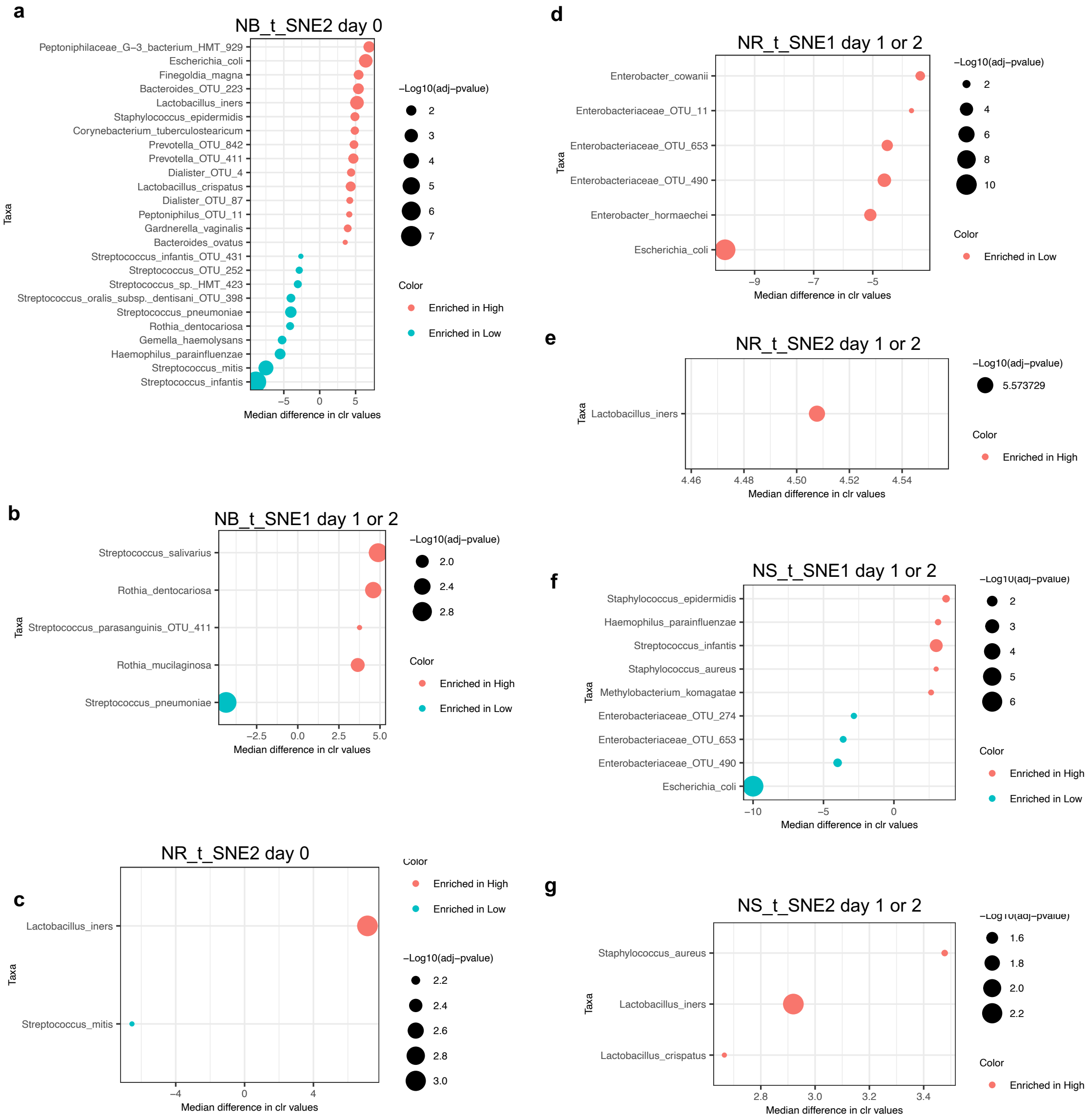

Fig. S5 Biological meaning of the t-SNE values of the neonatal microbiotas. The biological meaning of the t-SNE1 and t-SNE2 values in the neonatal microbiotas on different days was illustrated by testing the abundance differences between the microbiotas with high and low levels of the t-SNE values. Because no significant difference was detected, the biological meaning of some t-SNE values, i.e., t-SNE1 of NB on day 0, t-SNE2 of NB on day 1 or 2, and t-SNE1 of NR on day 0, are not shown.

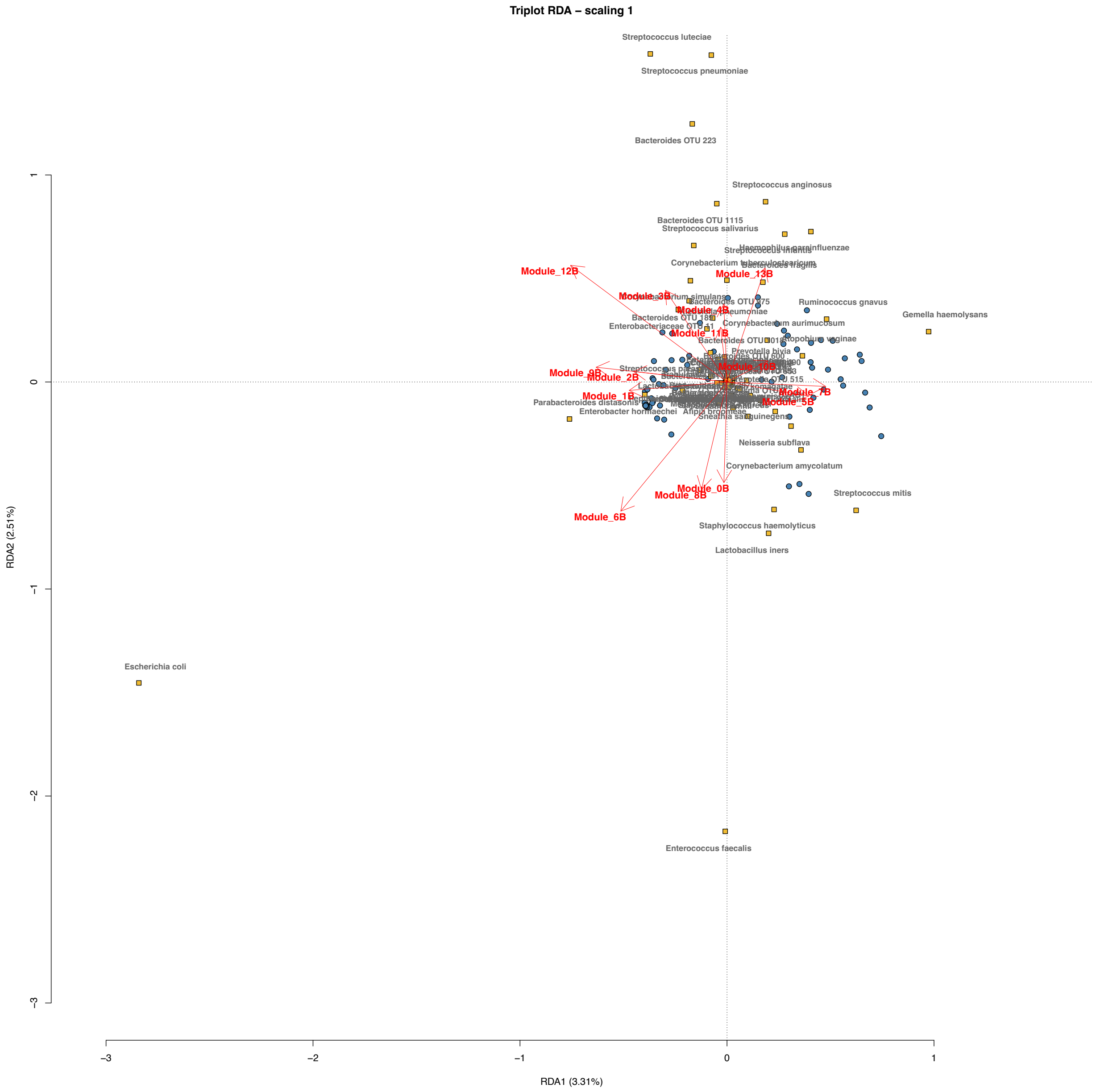

Fig. S6 The dbRDA test to show the association between the maternal modules and the composition of the NR microbiota on day 1 or 2.

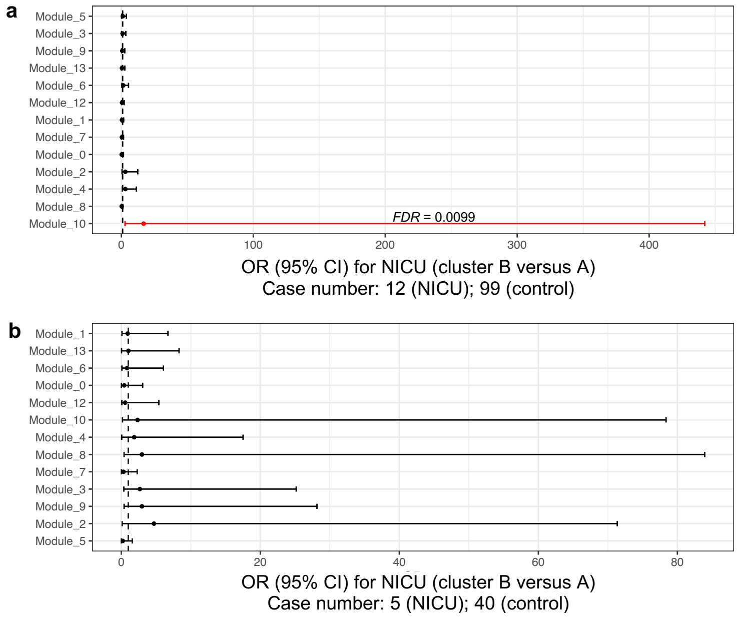

Fig. S7 The association between the maternal modules and the risk of NICU admission in term (a) and preterm (b) participants determined by the odds ratio analysis. Data are presented as risk ratios and upper and lower bounds of the estimate. Significant *FDR* values are shown.

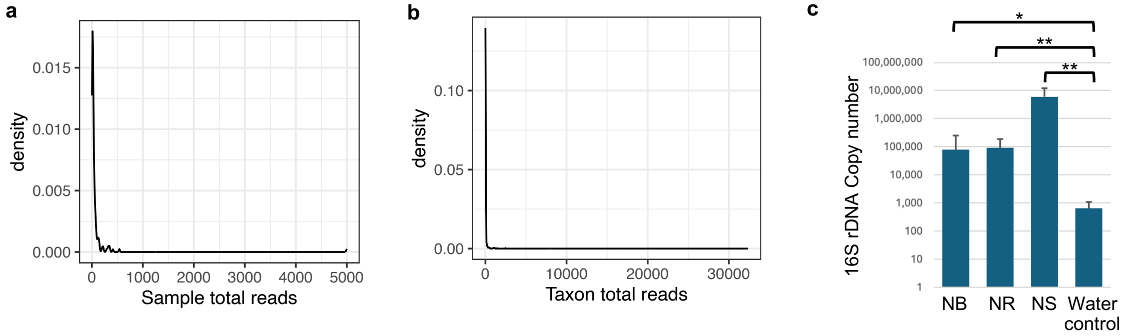

Fig. S8 The comparison between water controls and the neonatal microbiotas. The distributions of sample total reads **(a)** and taxon total reads **(b)** in 155 water controls are shown. **(c)** The bacterial biomass was quantified by q-PCR of 16S rDNA gene. The difference between the water controls and the neonatal microbiotas was determined by the two-sided Mann–Whitney U test. * P-value ≤ 0.05 and ** P-value ≤ 0.01.

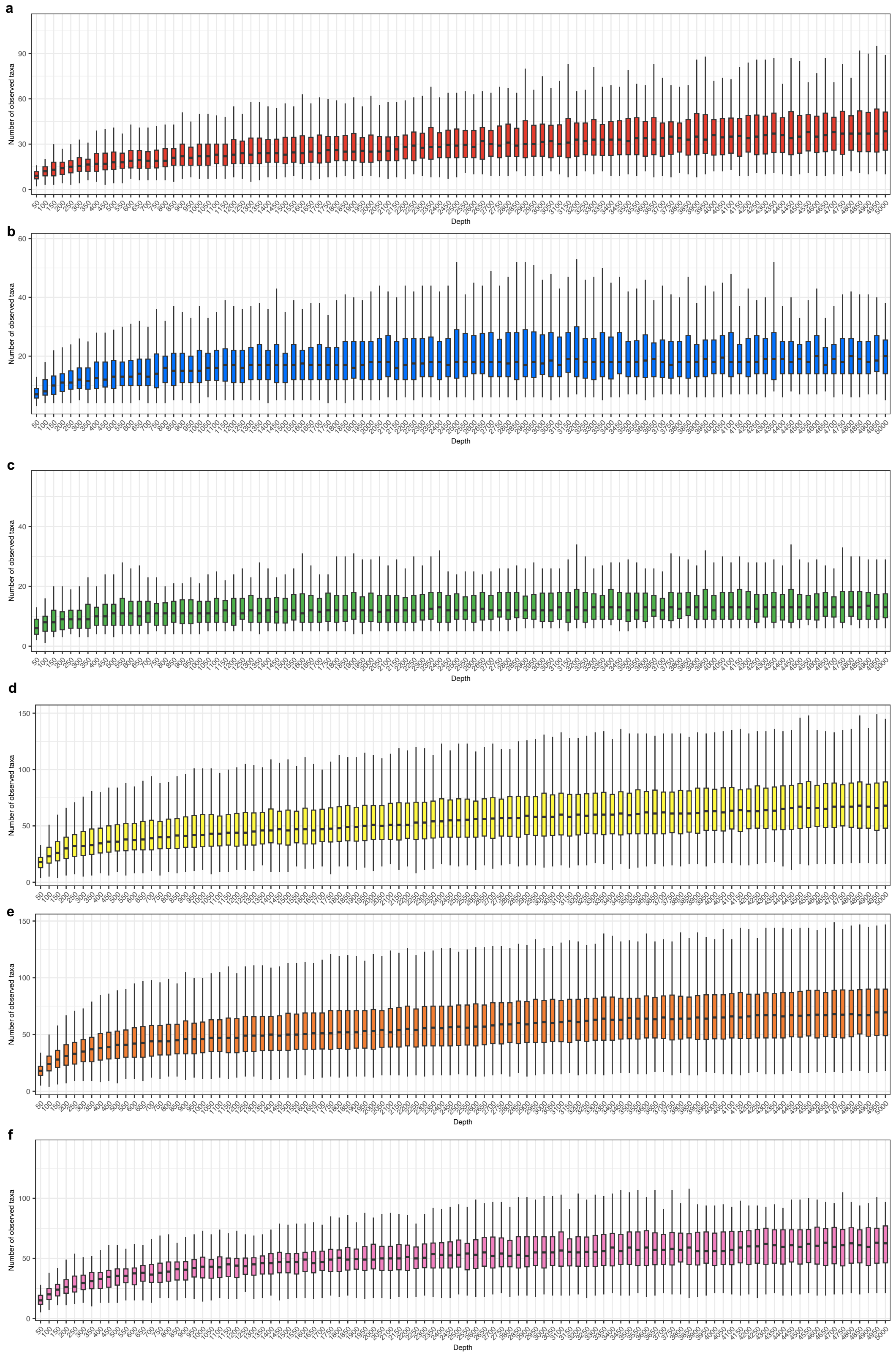
a
b
c
d
e
f
Number of observed taxa
Depth

Fig. S9 Rarefaction curves for the number of observed taxa in the NB (a), NR (b), NS (c), MB (d), MR (e), and MV (f) microbiotas. Lines in the boxplots represent maximum, 75% quantile, median, 25 quantile, and minimum values from top to bottom.

Supplementary Table 1 Tools used in this study.

**Name**	**Reference**	**Website**
R	The R Project for Statistical Computing	http://www.r-project.org/
V-Xtractor	[43]	https://github.com/carden24/V-Xtractor
USEARCH	[44]	https://www.drive5.com/usearch/
'vegan' package in R	(Jari Oksanen et al., 2020)	https://github.com/vegandevs/vegan
'Rtsne' package in R	[46]	https://github.com/jkrijthe/Rtsne
'ALDEx2' package in R	[31]	https://github.com/ggloor/ALDEx2_dev
'pheatmap' package in R	NA	https://github.com/raivokolde/pheatmap
'mice' package in R	[48]	https://github.com/amices/mice
'randomForest' package in R	[47]	https://github.com/cran/randomForest
Greengenes database	[41]	https://greengenes.secondgenome.com/
HOMD database	[42]	https://www.homd.org/
16S rRNA V1-V3 database generated in this study	This study	https://github.com/GregoryBucklab/Neonatal_microbiome_project
