# Supplementary material for: The association of maternal factors with the neonatal microbiota and health
Source: Nat Commun. 2024 Jun 19;15:5260. doi: 10.1038/s41467-024-49160-w (PMC11187136; doi:10.1038/s41467-024-49160-w)
Supplement: Supplementary file 3 — Description of Additional Supplementary Files [file 41467_2024_49160_MOESM3_ESM.docx]

**Supplementary Movie 1**

Description: Beta diversity changes of the neonatal buccal and rectal microbiotas within three days postpartum. A movie to show beta diversity changes of the neonatal buccal (red dots) and rectal (blue dots) microbiotas within three days postpartum. Beta diversity was quantified by Bray-Curtis distance and visualized by the t-distributed stochastic neighbor embedding plot. The maternal buccal (yellow dots), rectal (orange dots), and vaginal (pink dots) microbiotas on the last visit of pregnancy are visualized together with the neonatal microbiotas as a background.

**Supplementary Data 1**

Description: Metadata and other variables in this study.

**Supplementary Data 2**

Description: Association between every two maternal factors.

**Supplementary Data 3**

Description: Correlation among the maternal modules.

**Supplementary Data 4**

Description: Processed 16S rRNA reads profiles.

**Supplementary Data 5**

Description: Average reads per sample of each taxon in the 155 blank control samples and the neonatal samples.
