# Supplementary figures and images for: The association of maternal factors with the neonatal microbiota and health
Source: Nat Commun. 2024 Jun 19;15:5260. doi: 10.1038/s41467-024-49160-w (PMC11187136; doi:10.1038/s41467-024-49160-w)

## Slide 1
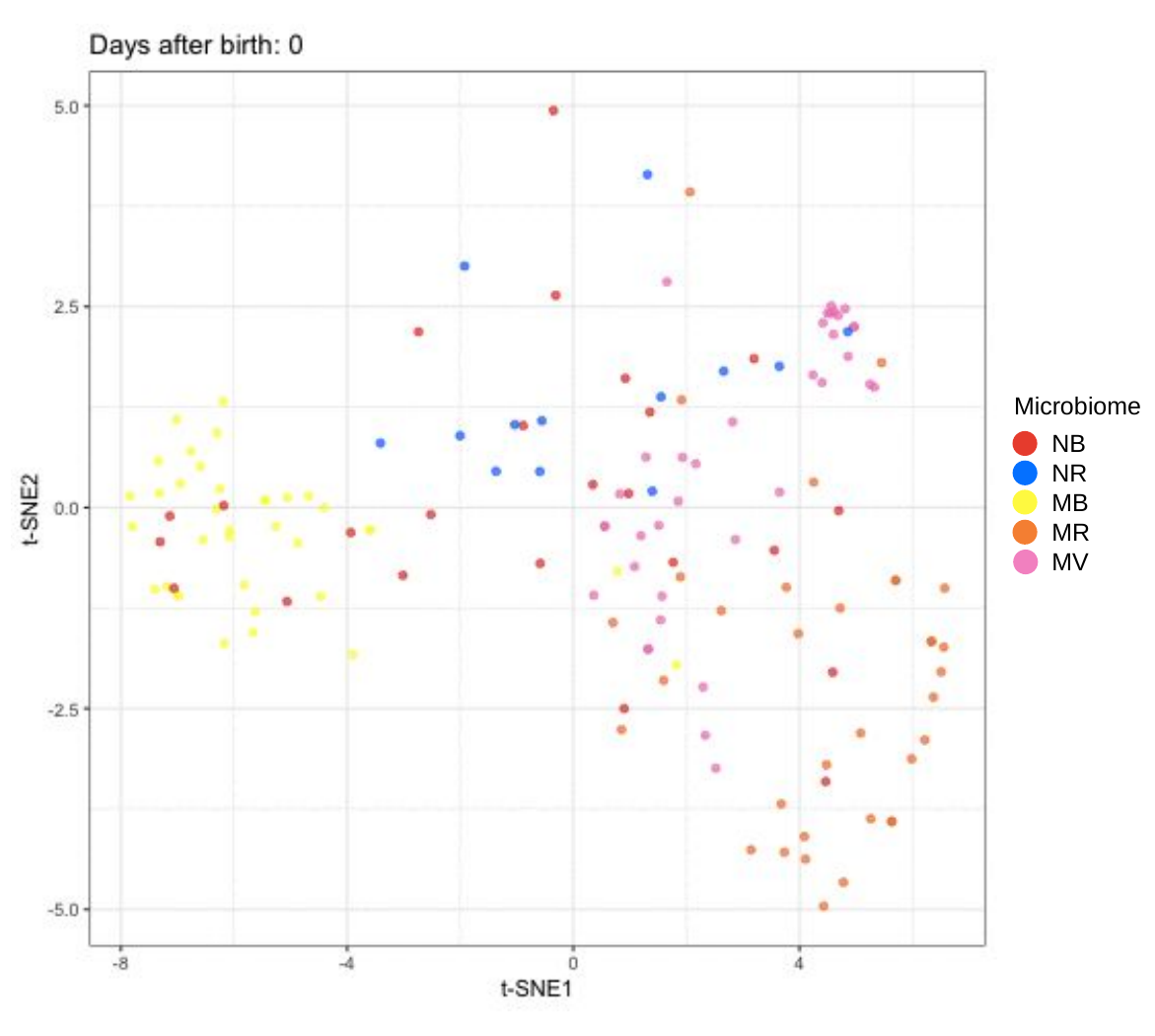

Microbiome
NB
NR
MB
MR
MV

Supplement: Supplementary file 9 — SI Movie 1 [file 41467_2024_49160_MOESM9_ESM.pptx]
