# Supplementary material for: The association of maternal factors with the neonatal microbiota and health
Source: Nat Commun. 2024 Jun 19;15:5260. doi: 10.1038/s41467-024-49160-w (PMC11187136; doi:10.1038/s41467-024-49160-w)
Supplement: Supplementary file 10 — Reporting Summary [file 41467_2024_49160_MOESM10_ESM.pdf]

Corresponding author(s): *Double-anonymous peer review submissions: write DAPR and your manuscript number here instead of author names.*

Last updated by author(s): *YYYY-MM-DD*

Reporting Summary

Nature Portfolio wishes to improve the reproducibility of the work that we publish. This form provides structure for consistency and transparency in reporting. For further information on Nature Portfolio policies, see our Editorial Policies and the Editorial Policy Checklist.

Statistics

For all statistical analyses, confirm that the following items are present in the figure legend, table legend, main text, or Methods section.

n/a	Confirmed	
☐	☒	The exact sample size (*n*) for each experimental group/condition, given as a discrete number and unit of measurement
☐	☒	A statement on whether measurements were taken from distinct samples or whether the same sample was measured repeatedly
☐	☒	The statistical test(s) used AND whether they are one- or two-sided<br>*Only common tests should be described solely by name; describe more complex techniques in the Methods section.*
☐	☒	A description of all covariates tested
☐	☒	A description of any assumptions or corrections, such as tests of normality and adjustment for multiple comparisons
☐	☒	A full description of the statistical parameters including central tendency (e.g. means) or other basic estimates (e.g. regression coefficient) AND variation (e.g. standard deviation) or associated estimates of uncertainty (e.g. confidence intervals)
☐	☒	For null hypothesis testing, the test statistic (e.g. *F*, *t*, *r*) with confidence intervals, effect sizes, degrees of freedom and *P* value noted<br>*Give P values as exact values whenever suitable.*
☒	☐	For Bayesian analysis, information on the choice of priors and Markov chain Monte Carlo settings
☒	☐	For hierarchical and complex designs, identification of the appropriate level for tests and full reporting of outcomes
☐	☒	Estimates of effect sizes (e.g. Cohen's *d*, Pearson's *r*), indicating how they were calculated

Our web collection on statistics for biologists contains articles on many of the points above.

Software and code

Policy information about availability of computer code

Data collection	All the codes for data collection are available on GitHub (https://github.com/GregoryBucklab/Neonatal_microbiome_project) with a DOI 10.5281/zenodo.11200086.
Data analysis	All the codes for data analysis are available on GitHub (https://github.com/GregoryBucklab/Neonatal_microbiome_project) with a DOI 10.5281/zenodo.11200086. All the tools used in this study are shown in Supplementary Table 1.

For manuscripts utilizing custom algorithms or software that are central to the research but not yet described in published literature, software must be made available to editors and reviewers. We strongly encourage code deposition in a community repository (e.g. GitHub). See the Nature Portfolio guidelines for submitting code & software for further information.

Data

Policy information about availability of data

All manuscripts must include a data availability statement. This statement should provide the following information, where applicable:

- Accession codes, unique identifiers, or web links for publicly available datasets
- A description of any restrictions on data availability
- For clinical datasets or third party data, please ensure that the statement adheres to our policy

Raw 16S rRNA sequences, cytokine data, and limited metadata of the Multi-Omic Microbiome Study-Pregnancy Initiative (MOMS-PI) project28,29 have been deposited in the HMP DACC (https://portal.hmpdacc.org). Controlled-access data for all subjects in the MOMS-PI project have been deposited at the National Center for Biotechnology Information’s controlled-access dbGaP (study no. 20280; accession ID phs001523.v1.p1; https://www.ncbi.nlm.nih.gov/projects/gap/cgi-

bin/study.cgi?study_id=phs001523.v1.p1) and the SRA under BioProject IDs PRJNA326441, PRJNA326442, and PRJNA326441. Other researchers can reproduce the reported analysis using the datasets listed above. All SI datasets and video are available on Figshare (DOI: https://doi.org/10.6084/m9.figshare.21905397).

Research involving human participants, their data, or biological material

Policy information about studies with human participants or human data. See also policy information about sex, gender (identity/presentation), and sexual orientation and race, ethnicity and racism.

Reporting on sex and gender	These initial studies were concerned with the vaginal microbiome. As such, all initial participants were biologically female. We did not collect self-reported gender or sexual orientation. A fraction (approximately 50%) of the neonates participating in the study were male.
Reporting on race, ethnicity, or other socially relevant groupings	The related information are described in SI Data 1 sheet 1. Matched case numbers are in SI Data 1 sheet 2.
Population characteristics	There are more than 200 population characteristics applied in this study. All the characteristics are described in SI Data 1.
Recruitment	Participants were enrolled in the Multi-Omic Microbiome Study: Pregnancy Initiative (MOMS-PI) and the Vaginal Human Microbiome Project under the umbrella of the National Institutes of Health Human Microbiome Project (https://commonfund.nih.gov/hmp). Women were enrolled in women's clinics associated with the Virginia Commonwealth University Health Center. Exclusion criteria included women incapable of understanding the informed consent or assent forms or who were incarcerated. Demographic, health histories, dietary assessments, and clinical data (e.g., gestational age, height, weight, blood pressure, vaginal pH, diagnosis, etc.) were collected. Clinical information about neonates was collected at birth (day 0) and at 24-48 hours (day 1) or 48-72 hours (day 2) after birth. Other exclusion criteria included: 1) inability to self-sample due to any reason; 2) significant vaginal bleeding; 3) ruptured membranes; 4) herpes lesions.
Ethics oversight	Study protocols were approved by the Virginia Commonwealth University institutional review board under protocols IRB# HM12169 or HM15527. Written informed consent or parental permission and assent were provided by participants or minors older than 15 years, respectively.

Note that full information on the approval of the study protocol must also be provided in the manuscript.

Field-specific reporting

Please select the one below that is the best fit for your research. If you are not sure, read the appropriate sections before making your selection.

[x] Life sciences [ ] Behavioural & social sciences [ ] Ecological, evolutionary & environmental sciences

For a reference copy of the document with all sections, see nature.com/documents/nr-reporting-summary-flat.pdf

Life sciences study design

All studies must disclose on these points even when the disclosure is negative.

Sample size	The number of available samples determined the sample size. The statistical power of sample size was calculated and is shown in Supplementary Data 2 and 6.
Data exclusions	16S rRNA sequencing profiles of the maternal and neonatal microbiomes with total reads less than 5,000 are not involved in this study. Metadata with more than 25% of missing values are not included in the multivariate testing on the association between metadata and the alpha and beta diversities of the neonatal microbiomes.
Replication	There is no replication in this study.
Randomization	All the participants are randomly selected. Exclusion criteria has been stated above.
Blinding	Investigators were blinded to group allocation during data collection and analysis.

Reporting for specific materials, systems and methods

We require information from authors about some types of materials, experimental systems and methods used in many studies. Here, indicate whether each material, system or method listed is relevant to your study. If you are not sure if a list item applies to your research, read the appropriate section before selecting a response.

Materials & experimental systems

n/a	Involved in the study
☒	☐ Antibodies
☒	☐ Eukaryotic cell lines
☒	☐ Palaeontology and archaeology
☒	☐ Animals and other organisms
☐	☒ Clinical data
☒	☐ Dual use research of concern
☒	☐ Plants

Methods

n/a	Involved in the study
☒	☐ ChIP-seq
☒	☐ Flow cytometry
☒	☐ MRI-based neuroimaging

Clinical data

Policy information about clinical studies

All manuscripts should comply with the ICMJEguidelines for publication of clinical research and a completedCONSORT checklist must be included with all submissions.

Clinical trial registration	This is not a clinical trial
Study protocol	This is not a clinical trial
Data collection	Women were enrolled in women's clinics associated with the Virginia Commonwealth University Health Center.
Outcomes	This is not a clinical trial

Plants

Seed stocks	n/a
Novel plant genotypes	n/a
Authentication	n/a
